# Epithelial Anion Transport as Modulator of Chemokine Signaling

**DOI:** 10.1155/2016/7596531

**Published:** 2016-06-12

**Authors:** Andrea Schnúr, Péter Hegyi, Simon Rousseau, Gergely L. Lukacs, Guido Veit

**Affiliations:** ^1^Department of Physiology, McGill University, Montréal, QC, Canada H3G 1Y6; ^2^Institute for Translational Medicine and 1st Department of Medicine, University of Pécs, Pécs 7624, Hungary; ^3^MTA-SZTE Translational Gastroenterology Research Group, Szeged 6720, Hungary; ^4^The Meakins-Christie Laboratories, Research Institute of the McGill University Health Centre, Montréal, QC, Canada H2X 2P2; ^5^Department of Biochemistry, McGill University, Montréal, QC, Canada H3G 1Y6; ^6^Groupe de Recherche Axé sur la Structure des Protéines (GRASP), McGill University, Montréal, QC, Canada H3G 1Y6

## Abstract

The pivotal role of epithelial cells is to secrete and absorb ions and water in order to allow the formation of a luminal fluid compartment that is fundamental for the epithelial function as a barrier against environmental factors. Importantly, epithelial cells also take part in the innate immune system. As a first line of defense they detect pathogens and react by secreting and responding to chemokines and cytokines, thus aggravating immune responses or resolving inflammatory states. Loss of epithelial anion transport is well documented in a variety of diseases including cystic fibrosis, chronic obstructive pulmonary disease, asthma, pancreatitis, and cholestatic liver disease. Here we review the effect of aberrant anion secretion with focus on the release of inflammatory mediators by epithelial cells and discuss putative mechanisms linking these transport defects to the augmented epithelial release of chemokines and cytokines. These mechanisms may contribute to the excessive and persistent inflammation in many respiratory and gastrointestinal diseases.

## 1. Introduction

**Figure 1:**
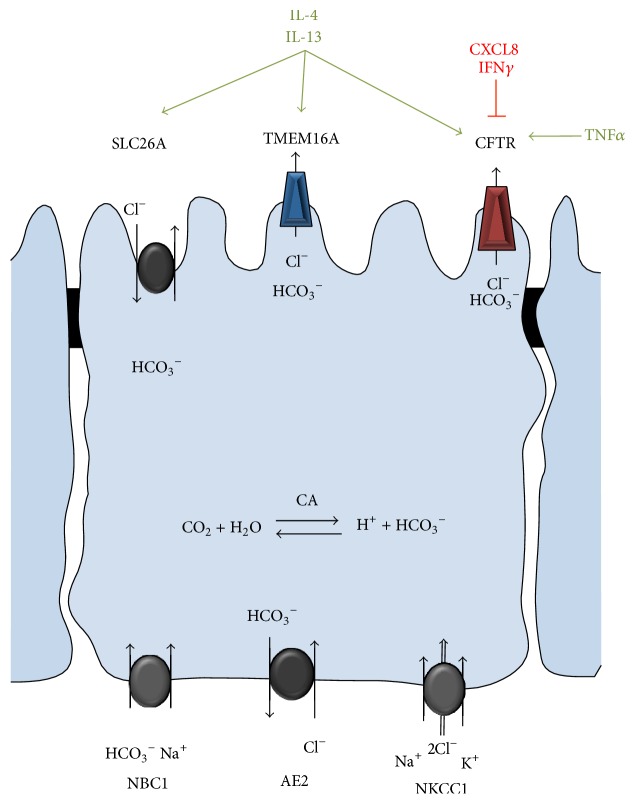
Schematic depiction of the anion transport pathways in secretory epithelia. Apical chloride (Cl^−^) and bicarbonate (HCO_3_^−^) efflux is mediated by CFTR and TMEM16A and probably members of the SLC26A family. Basolateral chloride and bicarbonate entry is conducted by cation cotransporters NKCC1 and NBC1, respectively. Alternatively chloride entry at the basolateral membrane is conducted by chloride-bicarbonate exchange via AE2. Carbonic anhydrase (CA) catalyzes the* de novo* formation of bicarbonate. Examples for the cytokine-mediated regulation of channel function or expression are indicated.

The ion and water homeostasis, mediated by channels and transporters at the apical and basolateral membranes of secretory epithelia that maintain a fluid compartment at the luminal surface, is essential for the function of different organ systems such as the airways, intestines, and pancreas [1, 2]. The most abundant anions, chloride and bicarbonate, are secreted through the apical cystic fibrosis transmembrane conductance regulator (CFTR) and calcium-activated chloride channel TMEM16A and anion exchangers of the SLC26A family. At the basolateral plasma membrane (PM) chloride and bicarbonate uptake are mediated by the sodium-potassium-chloride cotransporter (NKCC1) and sodium-bicarbonate cotransporters (NBC1), respectively [1] (Figure 1). Alternatively, basolateral chloride entry into the cell is conducted by the basolateral chloride-bicarbonate exchanger AE2 [3].

Secretory epithelial cells also play a pivotal role as part of the innate immune system. Besides providing a physical barrier by forming tight cell-cell contacts and a chemical barrier by secreting antimicrobial peptides and mucins, epithelial cells express a variety of pattern-recognition receptors that, when triggered, activate the expression and release of a number of chemokines and cytokines. These inflammatory mediators in concert with the production of reactive oxygen species (ROS) allow the epithelium to contribute to the host defense and to recruit specialized immune cells [4–7]. It is well established that the expression and function of ion channels and transporters, including the anion channel and transporters discussed in this review, are modulated by chemokines and cytokines [8–12]. Examples for these mechanisms include the inhibition and downregulation of the epithelial sodium channel (ENaC) by IL-4 and IL-13 [10, 13, 14], the IL-4 and IL-13-mediated increase in function and expression of CFTR [13], and TMEM16A [15] as well as pendrin (SLC26A4) [12, 16] and SLC26A9 [17]. The CFTR expression and activity are increased by TNF*α* [18] and decreased by IFN*γ* [19, 20] and its function is attenuated by CXCL8 via *β*2-adrenergic receptor (*β*2-AR) dependent mechanism [21] (Figure 1).

In contrast, little is known about the regulatory role of anion transport on cytokine, in particular chemokine, synthesis, and secretion of epithelial cell that serves as amplifier and regulator of inflammation in conditions like cystic fibrosis (CF), chronic obstructive pulmonary disease (COPD), asthma, cholestasis, and acute pancreatitis. The effects of aberrant anion transport activities in these diseases and putative mechanisms linking them to the augmented epithelial release of chemokines and cytokines are discussed in this review.

## 2. Diseases Exhibiting Defective Epithelial Anion Transport

### 2.1. Cystic Fibrosis

Cystic fibrosis (CF), the most common lethal hereditary disease in the Caucasian population, is caused by mutations in CFTR that lead to impaired anion conductance at the apical membrane of secretory epithelia [22–25]. More than 2,000 mutations in CFTR have been described that confer a range of molecular, cellular, and functional phenotypes [26, 27] and can be classified according to these complex phenotypes as recently reviewed [28]. Interestingly, mutants that are expressed at the apical membrane in densities comparable to the wild-type (WT) protein but are nonfunctional (e.g., G551D-CFTR) cause a similar CF disease severity as mutants with strongly reduced protein at the PM (e.g., ΔF508-CFTR) [27, 29], suggesting that the loss of CFTR function rather than its physical absence is required for the initiation of the disease.

CF is characterized by a multiorgan pathology mainly affecting the upper and lower airway, gastrointestinal and reproductive tract, and endocrine system [23, 25]. Pancreatic disease in CF has a high penetrance and the pancreas is the earliest intrauterine affected organ [30, 31]. Most CF patients (80–90%) suffer from pancreatic insufficiency (PI), a condition characterized by acinar atrophy, ductal irregularity, fibrosis, deficiency of exocrine pancreatic enzymes, and beta cell damage at late stages. Importantly, the level of pancreatic damage in CF is linked to the genotype [32, 33]. Patients that retain sufficient exocrine pancreatic function carry a small risk to develop pancreatitis [34, 35]. In the lung, imbalanced epithelial ion secretion and mucus thickening lead to decreased airway clearance and reoccurring bronchopulmonary infections that cumulate in tissue damage, the leading cause for morbidity and mortality in CF [36–39].

It is widely accepted that CF lung disease is associated with an excessive inflammatory response that is characterized by massive neutrophil recruitment into the lumen driven by augmented release of chemokines including CXCL8 by lung epithelial tissue. Once recruited and activated the neutrophils release antimicrobial peptides, neutrophil extracellular traps (NETs), reactive oxygen species, and proteases [40–42]. The sheer amount of neutrophil released serine proteases (neutrophil elastase (NE), proteinase 3, and cathepsin G) overwhelms the antiprotease production resulting in protease-antiprotease imbalance [43, 44]. Excessive amounts of NE in the airway-surface liquid in turn induce CXCL8 expression [45, 46], upregulate mucin production [47, 48], cleave antimicrobial peptides [49], and promote the degradation of CFTR [50] further perpetuating the inflammatory response that cumulates in progressive tissue damage. Despite their excessive infiltration into the CF lung, CF neutrophils fail to eradicate bacterial infections [41, 51]. A recent study shows that CF neutrophils exhibit abnormal degranulation responses and consequently impaired bacterial killing. Treatment of G551D-CFTR patient with the potentiator ivacaftor normalized the intracellular homeostasis and degranulation of their neutrophils, thus improved bacterial killing [52]. These findings and studies reporting reduced phagolysosomal chlorination in CF neutrophils [53, 54] suggest an intrinsic neutrophil function defect in CF. However, although hyperinflammation in CF is acknowledged, it is under debate whether the immunological aberrancy is directly caused by loss-of-CFTR-function or is rather the consequence of chronic bacterial infection (this controversy is reviewed in [42, 55–57]).

#### 2.1.1. Animal Models of CF

Animal models allow investigating the early pathogenesis of CF and examining how the lack of CFTR is linked to the host-defense defect of the lung. Several CF mouse models exist, including null [58, 59] and mutant form of CFTR [60–62] expressing animals. However, none of these models develops spontaneous severe lung disease, characteristic of human CF. Yet CF mice do secrete dramatically higher concentrations of inflammatory mediators upon bacterial challenge, including the chemokines keratinocyte chemoattractant (KC/CXCL1, the functional CXCL8 analog in mice) and the macrophage inflammatory protein 2 (mip-2/CCL3) that can be measured in the bronchoalveolar lavage (BAL) fluid [63, 64]. Concordantly, inhibition of CFTR in WT mice using the inhibitor-172 exacerbates the acute inflammatory response in an induced peritonitis model, including increased polymorphonuclear leukocyte infiltration and cytokine release [65]. The specificity of the inhibitor-172 however is questioned by other studies [66–68]. Transgenic mice that overexpress the *β*-subunit of the epithelial sodium channel (ENaC) in the airway, mimicking the increased sodium reabsorption found in human airway epithelia by many researchers [36, 37, 69], exhibit mucus obstruction, goblet cell metaplasia, increased BAL levels of the chemokines CXCL1 and CXCL2, neutrophilic inflammation, and poor bacterial clearance [70].

Since mice do not recapitulate all of the hallmarks of human CF, considerable efforts were made to create other animal models.* CFTR* ^−/−^ rats were recently created using DNA editing and the resulting animals show a reduced airway-surface liquid (ASL) height and submucosal gland hypoplasia [71].* CFTR* ^−/−^ and *CFTR* ^Δ*F*508/Δ*F*508^ pigs as well as* CFTR* ^−/−^ ferrets were generated by adeno-associated virus-mediated gene targeting and somatic cell nuclear transfer [72, 73]. CF pigs and ferrets develop spontaneous lung disease characterized by airway inflammation, airway remodeling, impaired mucosal clearance, and bacterial infection [74–76]. The clearance upon bacterial challenge in the lung of both animal models is impaired likely as a result of the decreased pH and therefore attenuated antibacterial properties of the ASL [77, 78]. The onset of pulmonary inflammation however differs between CF pigs and ferrets. The CXCL8 and TNF*α* levels were increased in the CF ferret BAL at birth, suggesting an inherent innate immunity defect [77]. In contrast neutrophil counts and CXCL8 levels were not augmented in the neonatal CF pig BAL [74, 76]. Intriguingly, the pancreas of newborn CF pig shows increased proportion of innate immune cells and inflammatory response without apparent infection [79]. Similarly, zebrafish that lacks functional CFTR shows progressive destruction of the exocrine pancreas accompanied by a significant increase in neutrophil infiltration [80]. Thus, upregulation of chemokine secretion that promotes neutrophil recruitment likely contributes to the early onset of inflammation in these animals.

#### 2.1.2. Human Studies

An early onset of excessive proinflammatory chemokine and cytokine release into the BAL of CF infants has been reported, but whether this precedes bacterial infection remains controversial [81–85]. CXCL8, neutrophils counts, and the concentration of free elastase were prominently increased in the BAL of CF patients in comparison to healthy controls [82–84, 86]. Other inflammatory mediators that are augmented in the CF lung include IL-6, IL-1*β*, and TNF-*α* [87], GM-CSF and G-CSF [88], IL-33 [89], HMGB1 [90], and the chemokine CCL18 [91]. Interestingly, a number of studies reported a decrease of the anti-inflammatory cytokine IL-10 in CF BAL [87, 92, 93]. By binding to the IL-10 receptor, IL-10 inhibits signaling pathways including the mitogen-activated kinase p38 and NF-*κ*B pathways that are associated with proinflammatory chemokine and cytokine expression.

Using tracheal or bronchiolar grafts from CF and non-CF human foetuses implanted into severe combined immunodeficient (SCID) mice Tirouvanziam and colleagues investigated the inflammatory state prior to any infection [94, 95]. Both upper and lower airway CF grafts showed increased CXCL8 expression and leukocyte infiltration, but the inflammation was more severe in the bronchiolar grafts leading to progressive tissue destruction. These results provide evidence that CF lung inflammation could arise solely from CFTR mutations in sterile environment.

#### 2.1.3. Cell Models

Increased CXCL8 expression in CF cell lines and CF primary human bronchial epithelial cells has been reported, both constitutively and following stimulation with* Pseudomonas aeruginosa* or TNF-*α*, implying CFTR-dependent alteration in the innate immune response [96–98]. However, elevated CXCL8 levels were not observed in other cellular models [99–101], while in some studies CFTR appeared to have a permissive role in CXCL8 secretion [102, 103]. The interpretation of these results is difficult since many derive from the comparison between clonally isolated CF and non-CF cell lines. Isolation of individual clones from heterologous cell populations, however, may favor unrepresentative phenotypes as clonal variations can exceed the underlying differences in the regulated CXCL8 secretion [67]. The genetic heterogeneity and possible epigenetic modification due to the repeated infection and inflammation in CF [104] restrict the comparability of primary human cells from CF patients and healthy individuals. However, a number of studies elegantly circumvented these limitations. Inhibition of CFTR function by continuous treatment with the inhibitor-172 in differentiated primary airway epithelial cells resulted in increased constitutive CXCL8 secretion that was further augmented by* Pseudomonas aeruginosa* infection [105]. CFTR inhibition by gene knockdown in the human intestinal epithelial cell lines Caco-2/15 and HT-29 [106] as well as in human the human airway epithelial cell line Calu-3 [107] similarly increased CXCL8 secretion. In another approach human CF respiratory epithelial models with the inducible expression of either CFTR or TMEM16A were generated. Induced expression of WT-CFTR or TMEM16A attenuated the proinflammatory chemokine CXCL8, CXCL1, and CXCL2 and cytokine IL-6 expression under air-liquid, but not liquid-liquid interface culture [67]. Likewise, transient lentiviral transduction that conferred the expression of WT-CFTR reduced CXCL8 secretion of primary human CF bronchial epithelia [67]. Interestingly, the expression of the nonfunctional G551D-CFTR or WT-CFTR in combination with channel inhibition failed to attenuate CXCL8 release, suggesting that CFTR-mediated anion transport activity at the apical PM is required to reduce the proinflammatory chemokine and cytokine secretion [67].

### 2.2. Chronic Obstructive Pulmonary Disease

Chronic obstructive pulmonary disease (COPD), encompassing two clinical phenotypes, chronic bronchitis, and emphysema, is attributed to the exposure to cigarette smoke and other environmental toxins [108, 109]. Genetic factors such as *α*1-antitrypsin gene mutations are also associated with disease [110]. COPD affects 64 million people and currently is the 4th leading cause of mortality worldwide [111]. COPD is characterized by persistent airflow limitation, chronic inflammatory responses, excessive mucus production, and recurrent cough, concordant to the CF lung phenotype. COPD shares several other clinical, immunological, and biochemical features with CF. Both are characterized by reduced mucociliary clearance, goblet cell metaplasia, chronic bacterial infections, excessive innate immune responses, and persistent neutrophilic inflammation. In comparison with CF, however, the inflammatory response in COPD includes more pronounced involvement of the adaptive immune system. An inverse correlation between the number of CD8+ T cells and the CD4+/CD8+ ratio to the lung function has been observed [112–114]. The CD8+ T cells release perforins and cause cytolysis and apoptosis of alveolar epithelial cells that is correlated to emphysema [115, 116].

The phenotypic similarity between CF and COPD raises the possibility that CFTR inactivation and/or downregulation may play an important role in the pathogenesis of COPD. Indeed, a number of studies support this hypothesis. (i) COPD disease severity is inversely correlated with CFTR protein expression [117]. (ii) CFTR function and expression are markedly attenuated in the respiratory epithelia of smokers and patients suffering from COPD [118, 119]. (iii) CFTR downregulation was observed upon cigarette smoke or cigarette smoke extract exposure of respiratory and intestinal epithelial cultures [120–122]. (iv) Cigarette smoke decreases the level of CFTR in lipid rafts in mice leading to defective autophagy [123]. Overall, these studies suggest that an acquired loss of CFTR function might contribute to the pathogenesis of COPD.

The inflammation characteristics of COPD also resemble that seen in CF. Cigarette smoke extract and acrolein, a smoke component, have been shown to increase the CXCL8 mRNA stability by activating the p38 MAPK/MK2 signaling pathway [124]. Gene expression levels of CXCL8, IL-6, CCL2, and CCL8 were elevated in both smokers and COPD patients [125]. This is consistent with the increased levels of CXCL1, CXCL8, and CCL2 in induced sputum and/or BAL fluid [109] and the augmented levels of CCL18 in the serum of COPD patients [126].

### 2.3. Asthma

Asthma affects approximately 300 million people worldwide. Between 5 and 15% are classified as severe asthmatics, specified by a state of heightened morbidity that is refractory to normal pharmacological treatment. Asthma is characterized by airway hyperresponsiveness (AHR), tissue remodeling as indicated by an increased proportion of airway smooth muscle in the airway wall [127], and chronic airway inflammation that together results in reversible airflow obstruction [128]. In severe asthmatics' sputa, besides hallmarks of allergic inflammation, there is increased neutrophil presence that is correlated with lowest lung function and worst asthma control [129]. The increase in smooth muscle mass leads to more airway constriction and contributes to the inflammatory milieu, including synthesis of the neutrophil chemoattractant CXCL8 in severe asthmatics [130]. Recent studies in the CFTR knockout pig [131] and using TMEM16A inhibitors in mouse and human airway smooth muscle [132, 133] support a role of these anion channels in smooth muscle hyperresponsiveness. Moreover, as described in the previous sections, links between defects in anion transport in epithelial cells and increased CXCL8 production may have interesting parallels in asthmatic airway smooth muscle to regulate the inflammatory milieu, particularly in severe asthmatics. The intricate connection between inflammation and anion transport in airway smooth muscle is reviewed elsewhere [134].

Current understanding suggests that beyond the smooth muscle phenotype, the airway epithelium is a key player in the pathology of asthma [135]. In addition to goblet cell hyper- or metaplasia and mucus hypersecretion [136–138], the asthmatic airway epithelium exhibits barrier defects [139, 140] and increased release of the chemokines and cytokines CXCL8, IL-6, GM-CSF, TGF-*β*1, IL-13, and IL-1*β* [141, 142] that are capable of triggering both allergic (Th2-mediated [143]) and neutrophilic (Th17-mediated [144]) inflammation. Moreover, the ratio of CXCL8 to the anti-inflammatory lipid mediator lipoxin A4 is increased in severe asthmatics, favoring proinflammatory environment that promotes neutrophil recruitment [145]. Eicosanoids synthesis occurs via the cellular cooperation between structural cells (such as epithelial cells) and immune cells (like neutrophils). Changes in substrate availability (arachidonic acid (AA) versus docosahexaenoic acid (DHA)) can skew the inflammatory response, with allergic individual showing lower levels of DHA [146]. In a murine model of asthma, the proinflammatory balance was reversed using the vitamin A-derivative fenretinide to favor DHA over AA [147].

Some of these epithelial defects are reminiscent of the changes observed in cystic fibrosis. For example, in CF imbalance increasing the AA/DHA ratio has also been observed [148] that can be ameliorated with fenretinide in a CF mouse model [149]. Indeed at least 50% of CF patients show signs of airway hyperresponsiveness and bronchodilator treatment-reversible airway obstruction [150–153], a condition referred to as “CF asthma” [154, 155]. Concordantly, studies found an increased percentage of asthma patients to be carriers of mutations in CFTR [156–158] and the nasal potential difference values of some of these patients scored in the CF range [157]. In addition to CFTR, the involvement of anion transport in the pathophysiology of asthma is suggested by aberrant expression of other epithelial anion transporters. (i) The expression of the anion exchanger pendrin (SLC26A4) that functions to import chloride from the ASL in exchange to bicarbonate and thiocyanate [159] is upregulated in the asthmatic airway epithelium likely as a consequence of Th2 cytokine (IL-4 and IL-13) signaling [160, 161]. Interestingly, pendrin overexpression alone is sufficient to trigger mucus hypersecretion, hyperresponsiveness, and elevated secretion of the chemokines CXCL1 and CXLC2 that in turn lead to neutrophil infiltration into the airway [162]. (ii) A polymorphism in the 3′ UTR of SLC26A9 that functions as CFTR-regulated chloride-bicarbonate exchanger or chloride channel [163–165] is associated with reduced protein expression and an increased risk for asthma [17]. (iii) The calcium-activated chloride channel TMEM16A expression in airway secretory cells is increased in asthma and correlates with mucus hypersecretion [132].

Taken together these evidences show an important role of the airway epithelium to regulate the inflammatory balance in asthma in addition to mucus hypersecretion. Thus, anion transport can significantly influence neutrophil recruitment via increased chemokine synthesis, which is particularly important for the more severe form of the disease.

### 2.4. Pancreatitis

Acute pancreatitis (AP) is a sudden inflammatory disorder of the pancreas. AP is one of the most common disorders requiring acute hospitalization among the GI diseases [166]. In its chronic form, pancreatitis (CP) can lead to decreased quality of life with continuous pain, diarrhoea, maldigestion, and consequently weight loss. Moreover in some cases, on the base of chronic inflammation, pancreatic ductal adenocarcinoma can develop. The human exocrine pancreas consists of two main secretory cell types: ductal and acinar cells. The two cell types, as a functional unit, interact closely to mediate the secretion of the pancreatic juice and have a conjoint role in the pathogenesis of pancreatitis [167]. Acinar cells synthetize, store, and secrete digestive zymogens that under physiological conditions are carried along the ductal system into the duodenum. AP originates from the premature activation of pancreatic zymogens, leading to the autodigestion of the pancreas that causes the recruitment of inflammatory cells. In addition, acinar cells produce a large amount of protons which are secreted with the pancreatic zymogens resulting in acidic fluid [168]. Ductal cells secrete a bicarbonate-rich, alkaline fluid that is required to neutralize the acinar acid load and carry the zymogens into the duodenum and that acts as a protective mechanism to prevent premature zymogen activation [169]. The ductal secretion contains up to 140 mM HCO_3_^−^ that is achieved by the concerted activity of anion transporters. Basolateral Na^+^-HCO_3_^−^ cotransporters, Na^+^/H^+^ exchangers, and H^+^-ATPases mediate HCO_3_^−^ uptake into the epithelial cells. On the luminal side CFTR in concert with SCL26A family Cl^−^/HCO_3_^−^ exchangers, specifically SLC26A6 (PAT1) and SLC26A3 (DRA), mediate the bicarbonate secretion. DRA serves as 2Cl^−^/1HCO_3_^−^ exchanger, whereas PAT1 functions with 1 : 2 Cl^−^ : HCO_3_^−^ stoichiometry that enables reaching the high bicarbonate concentration characteristic for the pancreatic juice [170, 171]. A direct molecular interaction between CFTR and SLC26A6 and A3 has been observed that results in reciprocal activation of both CFTR and the Cl^−^/HCO_3_^−^ exchangers [172, 173]. However, until now the exact role of SLC26A6 and SLC26A3 in the pancreatic ductal bicarbonate secretion remains controversial [174].

Etiological factors for AP mainly include biliary disease and alcohol consumption. Interestingly, these factors have been shown to impair the ductal bicarbonate transport. (i) Ethanol and its nonoxidative metabolites decrease the CFTR activity, mRNA expression, and its stability at the PM [175, 176]. (ii) At high concentration nonconjugated bile acids impair the ductal bicarbonate and fluid secretion by inhibiting ion transporters (Na^+^/H^+^ exchangers, Na^+^-HCO_3_^−^ cotransporters, and Cl^−^/HCO_3_^−^ exchangers) and by causing intracellular calcium overload that leads to mitochondrial damage and ATP depletion and could also affect CFTR [177–180]. (iii) Recent studies suggest that smoking increases the risk for pancreatitis [181–183]. Acrolein, a component of cigarette smoke, has a systematic inhibitory effect on CFTR activity [184] and therefore likely contributes to the CFTR inhibition in pancreatic ducts. (iv) Intra-acinar [185] or intraductal [186, 187] premature activation of trypsinogen to trypsin is a key event in the initiation of pancreatitis. Trypsin inhibits CFTR-mediated ion transport by a PAR-2 dependent pathway, thus leading to a decreased luminal pH that further accelerates the trypsinogen autoactivation [169]. (v) Lastly, heterozygous carriers of CFTR mutations have increased risk to develop pancreatitis [188–197]. The consequences of CFTR mutations on pancreatic function include high protein content and low-flow secretion that frequently results in obstruction and the destruction of the gland. Most CF patients with pancreatic insufficiency (PI) carry “severe” mutations on both alleles that result in the loss of exocrine function. In case “severe” CFTR mutation on one allele is paired with “mild” CFTR mutation on the second allele, most patients retain sufficient exocrine function for digestion, referred to as pancreas sufficient phenotype (PS) [198–200]. Because the exocrine function is necessary for the initiation of pancreatitis, the risk for pancreatitis is higher for those mutations manifesting in PS CF phenotype [201]. Interestingly, the CFTR variant R75Q, which has selective bicarbonate transport defect, is associated with pancreatitis but not classical CF. Overall, the aforementioned studies indicate that the alteration of pancreatic ductal anion transport plays a role in the development of pancreatitis, suggesting that CFTR could be a possible therapeutic target for AP [32].

AP results in local and systematic overproduction of inflammatory mediators [202]. Characteristic hallmark of the disease is the increased serum levels of CXCL8 and IL-6 as well as often IL-1 and TNF-*α*. CXLC8 and IL-6 serum levels correlate with disease severity [203, 204]. Similar to the CF airway epithelia, it is likely that the pancreatic epithelium itself is at least partly accountable for the augmented chemokine and cytokine production and therefore plays a role in the exacerbation of the pancreatic inflammation. This is supported by the expression of IL-33, IL-6, and CXCL8 in stressed acinar cells [205–208] as well as CXCL8 expression in pancreatic duct cells [209]. Furthermore, pancreatic ductal adenocarcinoma cell lines (CAPAN-1 and CAPAN-2) exhibit basal and LPS induced secretion of CXCL8 and IL-6 [210]. The results of these studies raise the intriguing possibility that impaired anion transport in the pancreatic ductal system could increase chemokine and cytokine secretion and therefore contribute to the progression of pancreatitis.

### 2.5. Cholestatic Liver Disease

Cholangiopathies, chronic progressive liver diseases (e.g., primary sclerosing cholangitis or primary biliary cirrhosis) characterized by chronic cholestasis, are frequently caused by drugs and antibiotics and are common in CF increasing the risk for mortality [211]. Cholestasis is bile flow stagnation that could result from either the failure of the bile formation in the liver cells (intrahepatic) or the blockade of the ductal secretory mechanism (extrahepatic), responsible for washing biliary secretion away from the hepatocytes. CFTR is expressed at the apical membranes of cholangiocytes and the gallbladder epithelium [212], where it acts as chloride channel. CFTR promotes chloride efflux to generate a luminal chloride gradient, the driving force for bicarbonate secretion via AE2 mediated chloride/bicarbonate exchange resulting in bicarbonate-rich choleresis [213, 214]. CFTR also regulates cholangiocyte secretion by stimulating ATP release into the ductal lumen. Luminal ATP triggers apical P2Y receptors resulting in calcium mobilization, which in turn activates TMEM16A-mediated bicarbonate secretion [215]. Accordingly, dysfunction of CFTR results in low ductal pH, thickened bile, impaired bile flow, and biliary obstruction [216, 217]. This condition leads to accumulation of hydrophobic bile acids and results in periductal inflammation [218, 219].

Similar to the lung epithelium, human biliary epithelial cells participate in the innate immunity. They express various Toll-like receptors and secrete chemokines and cytokines as well as antimicrobial factors [220]. Therefore these cells play a crucial role in the pathogenesis of different cholangiopathies, such as primary biliary cholangitis and biliary atresia [221–223]. Cultured biliary ductal epithelial cells secrete CXCL8 and CCL2 [224, 225] that drive neutrophil infiltration [226]. In addition several other chemokines, including CXCL1, CXCL5, CXCL6, CCL3, CCL4, CCL5, and CXCL10, are expressed by human biliary epithelial cells under both basal and stimulated conditions [227].

One study showed that a combination of proinflammatory cytokines inhibits bile ductular secretion through attenuating AE2 and CFTR function that is impeded by the inhibition of cAMP formation in isolated single biliary ductal units [228]. Other studies however highlighted the role of CFTR function in the innate immune response of the biliary epithelium. Dextran sodium sulphate induced colitis results in an induced inflammatory cholangiopathy in CF but not WT mice [229, 230]. Indeed, downregulation of CFTR in progressive familiar intrahepatic cholestasis and reduced AE2 in primary biliary cirrhosis patients have been reported [231, 232]. Additionally, non-CF-causing CFTR mutations have been associated with primary sclerosing cholangitis in some patients [233, 234], further supporting the role of CFTR in these diseases.

## 3. Putative Mechanisms Linking Aberrant Anion Transport to the Dysregulated Epithelial Chemokine Release

The inherited (CF) or acquired (COPD, asthma, pancreatitis, and cholestasis) loss of epithelial anion transport is correlated to the increased inflammation driven by the release of chemokines and subsequent immune cell infiltration of the respective organs (Table 1). While in some of the discussed diseases the causality of these events is incompletely established, there is little doubt that loss of epithelial CFTR function is the primary cause for CF. This raises the question how the lack of anion transport leads to the described disease pathogeneses or more specifically which chemical or physical alterations, caused by the lack of chloride, bicarbonate, or other anion transport, trigger the disease mechanisms.

### 3.1. Chloride Transport

**Figure 2:**
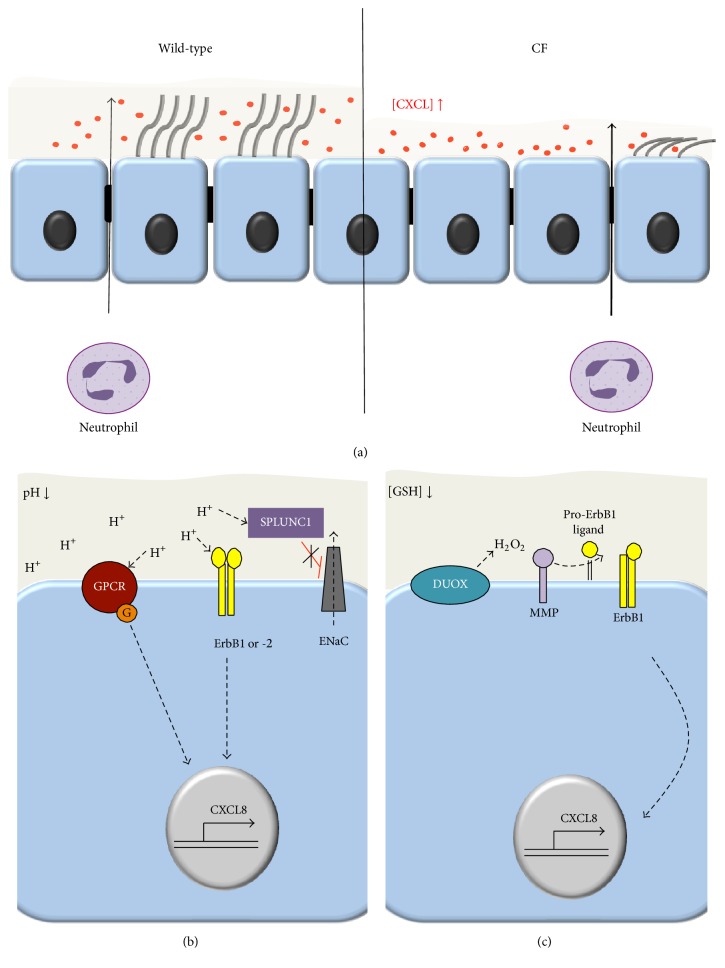
Putative mechanisms linking impaired anion transport to augmented epithelial chemokine signaling in secretory epithelia. (a) Low ASL in CF results in elevated concentrations of CXCL chemokines due to the reduced liquid volume in the luminal compartment. The increased concentration gradient enhances the neutrophil chemotaxis [250]. (b) Low ASL pH may directly activate chemokine expression by activating acid-sensing signaling pathways via proton-sensing GPCRs, ErbB1, and ErbB2 or the soluble protein SPLUNC1. (c) The decreased ROS buffering capacity of the ASL due to the reduced GSH concentration may promote hydrogen peroxide (H_2_O_2_) mediated metalloproteinase (MMP) activation. These in turn cleave pro-erbB1 ligands resulting in erbB1 activation, thus initiating signaling pathways that lead to the upregulation of chemokine expression [285].

The loss of CFTR-mediated chloride transport resulting in a decreased ASL height was proposed as primary causes of impaired mucociliary clearance, recurrent bacterial infection, and chronic neutrophilic lung infiltration in CF [36, 235, 236]. Reduced ASL height was observed in primary human CF airway epithelial cultures [236], human CF airway biopsies [237], CF ferret tracheas [77], and CF rat tracheas [71]. However, it is under debate whether CFTR inhibits the ENaC channel to prevent sodium hyperabsorption, which if this inhibition is lost contributes to the decreased ASL height [235, 238–240]. To counteract the reduced ASL height by rehydrating the mucus in CF, administration of nebulized hypertonic saline (HTS) was proposed. Administration of 7% HTS increased the mucociliary clearance and lung function and decreased the frequency of exacerbations [241, 242] (Table 2). In cell models hyperosmolarity increases the CXCL8 production by activating the p38 mitogen-activated protein kinase pathway [243, 244]. In contrast, in the sputum of CF patients HTS had either no effect [245, 246] or decreased CXCL8 levels and reduced the neutrophil chemotactic efficiency [247]. The latter observation is consistent with the HTS-mediated release of glycosaminoglycan bound CXCL8 that promotes its proteolytic cleavage and reduces the half-life and function of CXCL8 as neutrophil chemoattractant [247–249]. This leads to a reduced number of neutrophils in the CF sputum after HTS treatment [246]. A direct role of reduced ASL height in the augmented neutrophil chemotaxis in CF was suggested by a study of WT and ΔF508-CFTR tracheal epithelial cells [250]. Here,* Pseudomonas aeruginosa* infection increased the overall apical and basolateral release of the chemokines CXCL1, CXCL2, CXCL5, and CXCL10 to a similar extent in CF and WT epithelial cells. However, when the apical chemokine concentration was adjusted to the reduced ASL volume in CF, a significantly increased concentration gradient across the epithelial monolayer became apparent that explains the doubled transmigration of neutrophils across the CF monolayers [250] (Figure 2(a)).

### 3.2. Bicarbonate Transport

Lack of bicarbonate transport at the apical membrane of epithelia results in reduced pH of the epithelial lining fluid as indicated by measurements of the ASL, bile, or pancreatic fluid in CF [77, 78, 216, 251–254]. This pH difference might be attributed to the lack of CFTR-mediated bicarbonate transport [255, 256] or to the absence of CFTR-mediated regulation of the chloride/bicarbonate exchanger pendrin or other members of the SLC26 family [159, 163, 257]. The pH difference is augmented by the nongastric H^+^/K^+^ adenosine triphosphatase ATP12A activity that in human and pig mediates H^+^ transport into the ASL [258].

Reduced ASL bicarbonate and pH could provoke CF and CF-like phenotypes by multiple mechanisms. (i) Mucins packaging in goblet cell granules requires the presence of high intragranular calcium concentrations and acidic pH. Upon exocytosis, calcium chelation by bicarbonate and normal ASL pH facilitate mucin unfolding, prerequisite for their proteolytic cleavage and release from the cells surface [259, 260]. Impaired mucus detachment in CF pig submucosal glands and attenuated mucus release in CF mouse intestine highlight the importance of this mechanism [261–263]. In addition, the increased viscosity of mucins at acidic pH could contribute to the mucociliary clearance defect in CF [264]. (ii) Reduced ASL pH attenuates the bactericidal effect of antimicrobial peptides, key components of the innate immune response of the lung. pH-dependent activity was shown for lysozyme, lactoferrin, *β*-defensin-3, and LL-37 and a deleterious effect on their synergistic bactericidal properties upon pH-reduction was observed [78, 265]. (iii) The reduced ASL pH may also directly activate chemokine and cytokine expression and release by activating one or more of the following acid-sensing signaling pathways (Figure 2(b)). (a) Proton-sensing G-protein-coupled receptors, including GPR4, GPR65 (TDAG8), GPR68 (OGR1), and GPR132 (G2A), regulate the inflammatory response among other functions [266]. Upon activation by acidic pH GPR4 predominantly signals via the G_S_ pathway resulting in activation of cAMP formation [267, 268] and increased expression of inflammatory genes including chemokines (CXCL2, CXCL8, and CCL20), cytokines, adhesion molecules, NF-*κ*B pathway genes, and stress response genes [269]. GPR68, which is inactive at pH 7.8 and fully activated at pH 6.8, mainly acts through G_q_ [268]. It is expressed in airway epithelia and stimulates the phospholipase C activity as well as intracellular Ca^2+^ signaling and has been implicated in the acid-induced mucin5AC and IL-6 secretion [270, 271]. (b) The activity of the receptor tyrosine kinases ErbB1 and ErbB2 is increased by low extracellular pH [272], which can contribute to the increase in CXCL8 production [273]. (c) The short palate lung and nasal epithelial clone 1 (SPLUNC1) is a pH-sensitive regulator of ENaC and is unable to inhibit ENaC in the acidic CF airway environment [252]. Thus, the ensuing increased ENaC activity may result in decreased ALS volume [274] that can trigger augmented chemokine signaling as discussed above.

Aberrant mucin release and reduced bactericidal properties of the lining fluid could promote chronic bacterial infection that in concert with augmented chemokine and cytokine secretion would lead to the hyperinflammatory state seen in CF and other diseases. However, CFTR mutants like R75Q which have normal chloride but selective disruption of bicarbonate conductance do not cause classical CF [195] but are associated with recurrent acute and chronic pancreatitis [198].

### 3.3. Glutathione and Thiocyanate Transport

Another potential mechanism to perpetuate the imbalance in chemokine production in CF and perhaps other inflammatory disorders is oxidative stress arising from oxidant-antioxidant imbalance. Besides transporting chloride and bicarbonate, CFTR may serve as an efflux channel for reduced glutathione (GSH) [275, 276] and thiocyanate [277, 278]. The ASL concentration of GSH was estimated in the 275–430 *μ*M range in healthy individuals [279, 280]. In contrast in CF patients total GSH concentrations are reduced to 92 *μ*M but the concentration of the oxidised form (GSSG) was not affected by the absence of CFTR activity, thus shifting the redox potential from −175 mV to −128 mV [280]. This shift in redox potential may decrease the redox buffering capacity of the ASL and therefore affect the oxidation state of membrane proteins that could induce downstream proinflammatory signaling [281, 282]. However, it is not well understood how differences in extracellular GSH could result in aberrant gene expression. Increased or insufficiently buffered hydrogen peroxide production at the apical membrane of CF epithelia by NADPH oxidases, especially by the dual oxidase 1 (DUOX1) [283, 284], may increase metalloproteinase-mediated release of ligands that activate the ErbB1 signaling pathway resulting in augmented CXCL8 production [273, 285] (Figure 2(c)). This is consistent with the protective role of extracellular GSH supplementation upon infection with* Pseudomonas aeruginosa* in CF cells that reduced the release of CXCL8 and IL-6 [97, 286].

Direct GSH supplementation was explored as a therapeutic approach for CF as early as 1985. In this study the authors demonstrated that aerosol administered GSH led to an increase in BAL GSH and GSSG levels and decrease in reactive oxygen species [287]. While this and other initial studies were promising, showing an increase in BAL GSH level and improved lung function [288], the improvement could not be confirmed in a recent phase 2b trial [289]. Antioxidant treatment with N-acetylcysteine (NAC) that has a weak antioxidant character but predominantly acts through the replenishment of GSH in deficient cells [290] was explored as alternative to GSH. Initial short-term studies (≤3 month) did not report a significant improvement in CF lung function [291, 292]. Interestingly, administering high doses of NAC three times daily reduced NE, neutrophil counts, and CXCL8 in the sputum of CF patients [293]. A recent phase 2 trial reports a significant relative increase in FEV_1_ by 4.4% after 24-week treatment, which is predominantly due to stabilization of the lung function in the treatment group while it declined in the control group [294] (Table 2).

Thiocyanate acts both as an antioxidant [295] and precursor for the bactericidal hypothiocyanite [296]. Reduced level of thiocyanate in the epithelial lining fluid has been observed in CF cells, CF mice [297], and CF patients [298]. Restoring the thiocyanate levels in *β*-ENaC overexpressing mice, a model for CF [70], using nebulized thiocyanate decreased the bacterial burden after* Pseudomonas aeruginosa* infection and attenuated neutrophil infiltration and reduced the levels of the chemokine CXCL1 and cytokines IL-6 and TNF-*α* in the BAL [299].

## 4. Conclusion

The discussed mechanisms, linking attenuated anion transport to the inflammatory state in CF and other diseases, are not mutually exclusive. Therefore it is likely that symptomatic correction of any single one of these transport defects will result in only minor benefit. This is evident by the results of the clinical trials using HTS to rehydrate the mucus in CF that resulted in only a modest improvement in lung function [241, 242] and using inhaled GSH to increase the ASL antioxidant level that was not beneficial [289] (Table 2). Recent advances to correct the underlying defect in CF by folding correctors and gating potentiators of CFTR are promising, and drugs targeting a subset of CF-causing mutations are approved [25, 28]. These compounds might be beneficial for other diseases with an acquired loss of CFTR function phenotype like, for example, COPD. Treatment with the gating potentiator ivacaftor increased the WT-CFTR activity in cigarette smoke exposed primary human bronchial epithelia and partially reverted the smoke-induced mucociliary clearance defect [300]. The University of Alabama recently announced a phase I clinical trial with ivacaftor in COPD patients [301]. Considering the limited efficacy of this potentiator to increase the open probability of WT-CFTR [302] and the documented downregulation of CFTR expression in COPD [117, 122], stabilizing the protein with a corrector drug would be another promising strategy.

Alternative ion channels like TMEM16A or SLC26A9 may compensate for CFTR dysfunction [303]. For TMEM16A this was attempted using the stable UTP analog denufosol that stimulates P2Y-receptor-induced cytoplasmic calcium signaling and thus channel activity [304] (Table 2). This approach was unsuccessful [305], likely due to the rapid desensitization and internalization of the P2Y-receptor [67, 306–308]. These limitations could be overcome by the activator F that probably activates TMEM16A allosterically without triggering cytosolic calcium signaling [309] and that was shown to reduce CXCL8 secretion in a CF cell model [67]. TMEM16A is widely expressed [132], including in the airway smooth muscle where it is implicated in the hyperresponsiveness in asthma [133, 310]. To target this channel for therapy therefore requires the identification and exploitation of an epithelial cell specific splice isoform [311] or regulatory mechanism.

## Figures and Tables

**Table 1:** Anion transport activity changes and proinflammatory chemokine and cytokine secretion in different diseases.

	Disease	Transporter/channel function	Augmented chemokines/cytokines
Airway system	CF	CFTR ↓	CXCL8, CXCL1, CXCL2, CCL3, CCL18, IL-1*β*, IL-6, IL-33, TNF*α*, GM-CSF, G-CSF, HMGB1
	COPD	CFTR ↓	CXCL8, CXCL1, CCL2, CCL8, CCL18, IL-6
	Asthma	CFTR^*∗*^ SLC26A4 ↑ TMEM16A ↑ SLC26A9 ↓	CXCL8, IL-1*β*, IL-6, IL-13, TGF-*β*1, GM-CSF

Gastrointestinal system	Pancreatitis	CFTR^*∗*^ CFTR ↓ SLC26A3/A6 ↓	CXCL8, IL-1, IL-6, TNF-*α*
	Cholestatic liver diseases	CFTR^*∗*^ CFTR ↓ AE2 ↓	CXCL8, CXCL1, CXCL5, CXCL6, CXCL10, CCL2, CCL3, CCL4, CCL5,

CFTR^*∗*^: carriers of CFTR mutations have an increased risk to develop disease.

**Table 2:** Clinical trials for CF therapy indirectly targeting the lack of CFTR-mediated anion transport in the lung by ion-replacement or activation of alternative anion channels.

Therapeutic approach	Compound	Type of administration	Result	References
Mucus rehydration	Hypertonic saline	Nebulized	Modest increase in lung function	[241, 242]

Antioxidant treatment	GSH	Nebulized	Small or no effect on lung function	[288, 289, 312]
	N-acetylcysteine	Oral	Modest increase in lung function	[291, 294]

TMEM16A function	Denufosol	Inhaled	Small or no effect on lung function	[305, 313]
